# RhoA-ROCK Inhibition Reverses Synaptic Remodeling and Motor and Cognitive Deficits Caused by Traumatic Brain Injury

**DOI:** 10.1038/s41598-017-11113-3

**Published:** 2017-09-06

**Authors:** Shalaka Mulherkar, Karen Firozi, Wei Huang, Mohammad Danish Uddin, Raymond J. Grill, Mauro Costa-Mattioli, Claudia Robertson, Kimberley F. Tolias

**Affiliations:** 1 Department of Neuroscience, Baylor College of Medicine, Houston, TX 77030 USA; 2 Memory and Brain Research Center, Baylor College of Medicine, Houston, TX 77030 USA; 3 Department of Integrative Biology and Pharmacology, University of Texas Medical School at Houston, Houston, TX 77030 USA; 4 Department of Neurosurgery, Baylor College of Medicine, Houston, TX 77030 USA; 5 Verna and Marrs McLean Department of Biochemistry and Molecular Biology, Baylor College of Medicine, Houston, TX 77030 USA; 6 Present Address: The Solomon Snyder Department of Neuroscience, Johns Hopkins University School of Medicine, 733N. Broadway, Baltimore, MD 21205 USA; 7 Present Address: Department of Neurobiology and Anatomical Sciences, University of Mississippi Medical Center, Jackson, MS 39216 USA

## Abstract

Traumatic brain injury (TBI) causes extensive neural damage, often resulting in long-term cognitive impairments. Unfortunately, effective treatments for TBI remain elusive. The RhoA-ROCK signaling pathway is a potential therapeutic target since it is activated by TBI and can promote the retraction of dendritic spines/synapses, which are critical for information processing and memory storage. To test this hypothesis, RhoA-ROCK signaling was blocked by *RhoA* deletion from postnatal neurons or treatment with the ROCK inhibitor fasudil. We found that TBI impairs both motor and cognitive performance and inhibiting RhoA-ROCK signaling alleviates these deficits. Moreover, RhoA-ROCK inhibition prevents TBI-induced spine remodeling and mature spine loss. These data argue that TBI elicits pathological spine remodeling that contributes to behavioral deficits by altering synaptic connections, and RhoA-ROCK inhibition enhances functional recovery by blocking this detrimental effect. As fasudil has been safely used in humans, our results suggest that it could be repurposed to treat TBI.

## Introduction

Traumatic brain injury (TBI) is a leading cause of death and disability. Individuals with TBI often suffer from debilitating cognitive, motor and behavioral impairments, including learning and memory loss, years after the initial injury^1^. Unfortunately, effective strategies for the treatment of TBI remain elusive. The underlying cause of TBI-induced deficits involves cell death and disruption of neuronal circuits in brain areas such as the hippocampus, which is critical for learning and memory^2, 3^. While some functional recovery occurs post-injury as a result of regenerative processes such as axonal and dendritic growth and the formation of dendritic spines (the primary sites of excitatory synapses), it is often limited due to the hostile growth environment of the adult central nervous system (CNS)^4, 5^. Thus, a greater understanding of the mechanisms that promote neural protection and repair is needed to develop novel therapeutic strategies to treat TBI.

A promising approach for enhancing recovery following TBI involves modulating the activity of small Rho-family GTPases^6, 7^. Rho GTPases are key cytoskeletal regulators that control CNS development and remodeling by directing diverse processes including cell morphogenesis, migration, proliferation, and survival^8, 9^. In neurons, the Rho GTPase Rac1 promotes the growth of axons and dendrites and the formation and maintenance of spines/synapses, whereas RhoA induces axonal and dendritic retraction and spine/synapse loss^10^. RhoA also plays an important role in CNS injury; it is robustly upregulated and activated following both brain and spinal cord injury, resulting in growth cone collapse and failed axon regeneration^11–15^. Excessive RhoA activation may also be responsible for the significant spine and synapse loss observed after TBI^10, 16^, which could contribute to deficits in information processing and memory storage. Notably, inhibiting RhoA or its key downstream effector Rho kinase (ROCK) in rodent models of spinal cord injury reduces inflammation and neuronal apoptosis and accelerates axonal regrowth, enhancing functional recovery^17^. Moreover, blockade of this pathway improves spatial and working memory in aged rats and rat models of Alzheimer’s disease and promotes recovery of neurological function in human patients following ischemic stroke^18–20^. While mounting evidence suggests that the RhoA-ROCK signaling pathway is a promising therapeutic target for CNS injuries including spinal cord injury and ischemia, it is not known whether inhibiting RhoA signaling will enhance neural protection and repair and restore cognitive function after TBI. Further, the mechanisms by which suppressing RhoA-ROCK signaling might protect neurons against the deleterious effects of TBI are not clear.

In this study, we show that TBI causes substantial motor, learning, and memory impairments in adult mice, which are alleviated by blocking RhoA-ROCK signaling via *RhoA* deletion from postnatal neurons or by treating mice with the ROCK inhibitor fasudil. While inhibiting RhoA-ROCK signaling does not impede cortical tissue loss at the contusion site, it does prevent TBI-induced dendritic spine remodeling and mature spine/synapse loss on hippocampal pyramidal neurons. Together, our data suggest that TBI elicits pathological spine remodeling that likely contributes to behavioral deficits due to loss and/or alteration of established synaptic connections, and that inhibiting RhoA-ROCK signaling enhances functional recovery by blocking this detrimental effect.

## Results

### Conditional ablation of *RhoA* from mouse postnatal forebrain neurons

**Figure 1:**
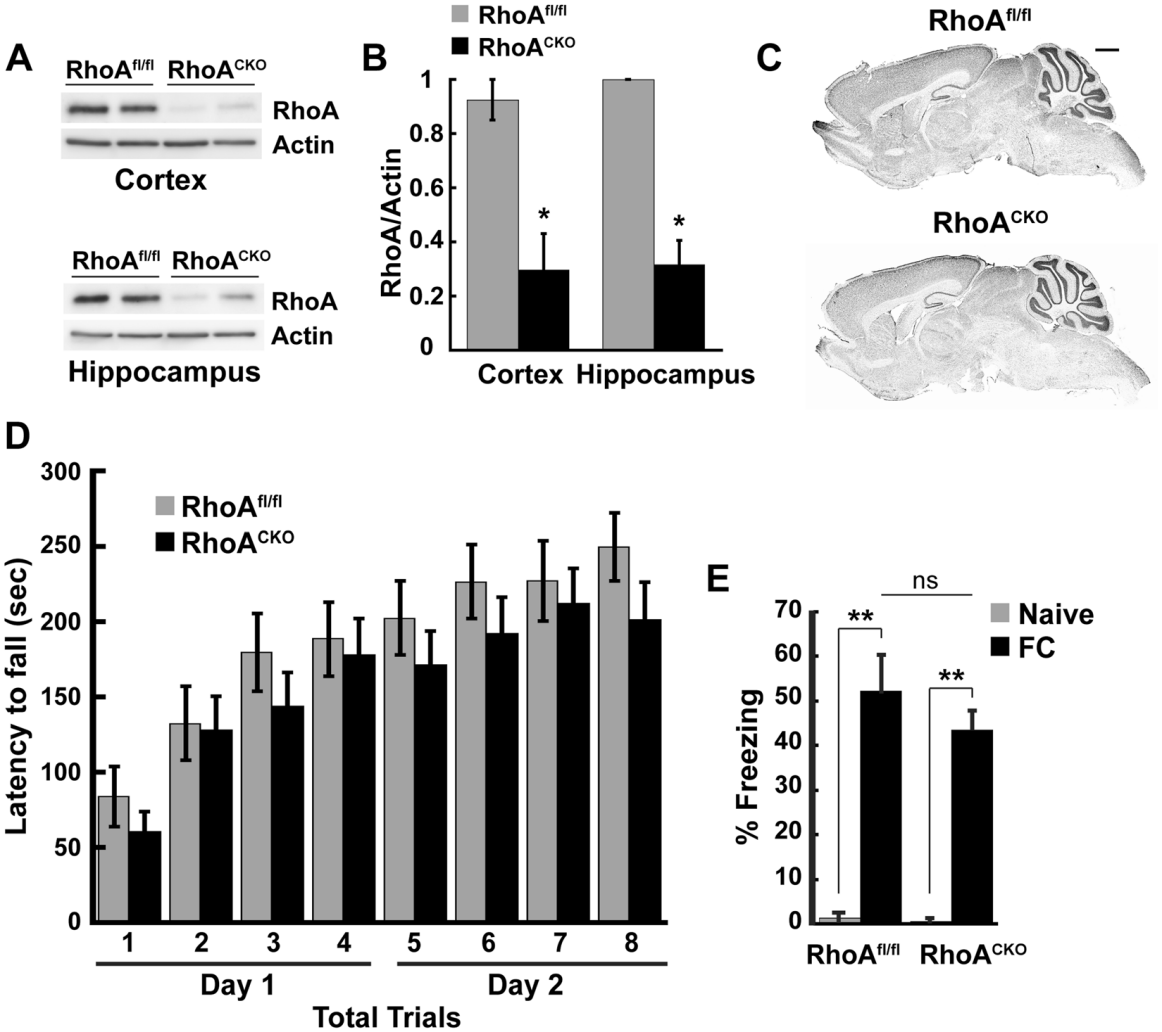
RhoA^CKO^ mice exhibit normal brain development and behavior. (**A**) Cortical and hippocampal homogenates from 3-month old RhoA^fl/fl^ and RhoA^CKO^ mice were immunoblotted for RhoA or actin (loading control). Representative blots show decreased RhoA levels in mutant mice. (**B**) Quantification of RhoA expression levels in control and RhoA^CKO^ mice. Protein bands were quantified using NIH Image J software and normalized using actin as control. (N = 3 mice/genotype, *p < 0.05, Student’s t-test). (**C**) Representative Nissl stained 50 μm sagittal brain sections from adult RhoA^fl/fl^ and RhoA^CKO^ mice reveal that mutant mice have grossly normal brain structures (N = 3). Scale bar = 500 μm. (**D**) RhoA^fl/fl^ and RhoA^CKO^ mice were tested on an accelerating rotarod for two days (4 trials per day) and their motor performance was compared. No significant difference was detected between RhoA^fl/fl^ and RhoA^CKO^ mice. N = 11 mice/genotype. Data were analyzed by two-way ANOVA with repeated measures. (**E**) RhoA^fl/fl^ and RhoA^CKO^ mice were subjected to fear conditioning. Mice from both groups displayed similar minimal freezing before training (Naive) and robust freezing 24 hr after training (FC), indicating that long-term memory in RhoA^CKO^ mice is normal. N = 11–17 mice/genotype. Figures show mean ± SEM; *p < 0.05, **p < 0.01, ns = not significant, ANOVA, followed by Tukey post hoc analysis.

CNS trauma induces the upregulation and activation of RhoA after injury, which promotes apoptotic cell death and restricts neuronal repair^12, 13, 21^. Inhibiting RhoA signaling could therefore minimize the deleterious effects of TBI and enhance functional recovery. To test this hypothesis, we blocked RhoA signaling by genetically ablating *RhoA* in mice. Since our group and others have shown that embryonic deletion of *RhoA* in neuroprogenitor cells disrupts CNS development^22–24^, we crossed RhoA^fl/fl^ mice with CamKIIα-Cre mice to ablate *RhoA* in the postnatal brain [(conditional knockout (KO)]. CamKIIα-Cre mice express Cre recombinase in postmitotic neurons in the forebrain, including cortex and hippocampus, with peak expressions between postnatal day (P) 21–90^25^. To confirm RhoA loss, we performed western blot analyses on hippocampal and cortical brain lysates prepared from control (RhoA^fl/fl^) and RhoA^fl/fl^; CamKIIα-Cre (RhoA^CKO^) mice (Fig. 1A). While RhoA protein levels in RhoA^CKO^ mice were similar to that of control mice at 1 month (Fig. S1A,B), they were significantly reduced by 3 months of age (Fig. 1A,B), consistent with late neuronal deletion of *RhoA*. Complete loss of RhoA was not expected as RhoA is also expressed in non-neuronal cells including glia^26^ and Cre is only expressed in post-mitotic excitatory neurons^25^. Initial characterization of the RhoA^CKO^ mice revealed that they were viable, fertile, and their brain structures were grossly normal (Figs 1C and S1C). We also evaluated the baseline motor and cognitive function of RhoA^CKO^ mice by subjecting them to different behavioral tests. To assess locomotor performance, we placed control RhoA^fl/fl^ and RhoA^CKO^ littermates on an accelerating rotarod and recorded their latency to fall (4 trials per day for 2 days). We found that RhoA^CKO^ mice performed similar to control mice on the rotarod (Fig. 1D), suggesting that their motor coordination and balance were relatively normal. To examine cognitive function, RhoA^fl/fl^ and RhoA^CKO^ mice were subjected to contextual fear conditioning, a robust form of hippocampal-dependent associative learning that is rapidly acquired and long lasting^27^. Contextual fear conditioning was performed by pairing a context (test chamber - conditioned stimulus) with a mild foot shock (aversive unconditioned stimulus), and then assessing memory by measuring the fear response (i.e. freezing behavior) of mice in the test chamber before fear conditioning (Naive) and 24 h after fear conditioning (FC), as previously described^28, 29^. Control and RhoA^CKO^ mice both displayed minimal baseline freezing behavior before conditioning and equivalent robust freezing behavior after conditioning (Fig. 1E), suggesting that contextual fear memory is normal in RhoA^CKO^ mice. Thus, silencing RhoA signaling in forebrain neurons late in development does not noticeably disrupt brain structure or function. Therefore, RhoA^CKO^ mice are a good model system for determining the specific roles of neuronal RhoA in TBI recovery.

### Deletion of neuronal RhoA does not prevent cortical tissue loss but does promote recovery of motor function after TBI

**Figure 2:**
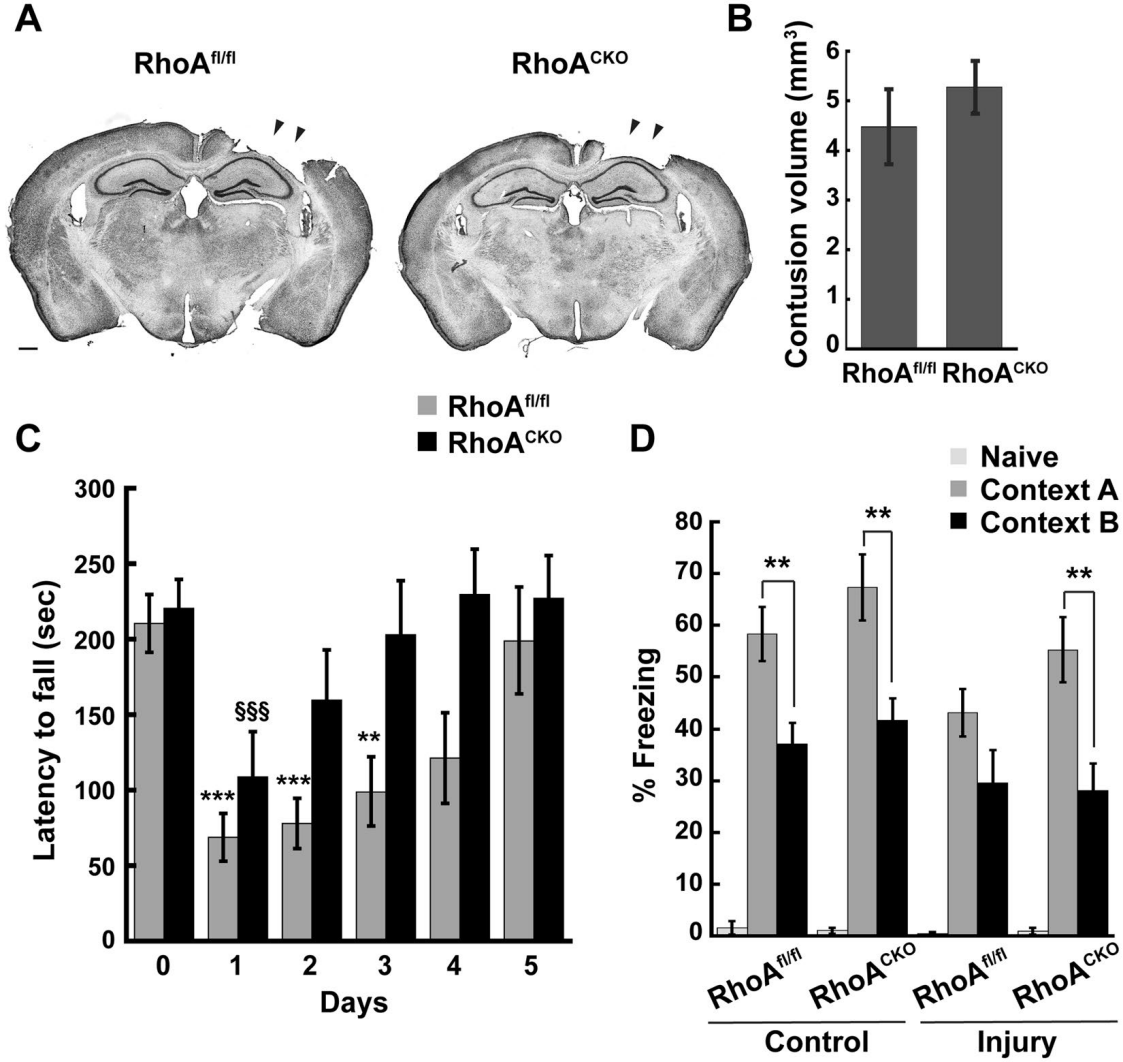
Loss of neuronal RhoA preserves motor and cognitive function after TBI. (**A**) RhoA^fl/fl^ and RhoA^CKO^ adult male mice were subjected to moderate CCI and brains were collected 14 days after injury. Brains were sectioned, Nissl stained, and imaged, and contusion volumes were calculated using NIH Image J. Scale bar = 500 μm. (**B**) Quantification of contusion volume revealed no difference between the two genotypes. Data were compared using Student’s t-test, N = 13–15 mice/genotype. (**C**) Pre-trained RhoA^fl/fl^ and RhoA^CKO^ mice were tested for motor performance on an accelerating rotarod prior to injury (day 0) and 5 consecutive days post-injury. RhoA^CKO^ mice recovered motor function faster than RhoA^fl/fl^ mice. Statistical significance was determined with two-way ANOVA with repeated measures. To compare performance of individual mouse groups on different days relative to day 0 (pre-TBI), we used one-way ANOVA with repeated measures. Significance indicated as: RhoA^fl/fl^ mice (*) or RhoA^CKO^ mice (§). N = 8 mice/genotype. (**D**) RhoA^fl/fl^ and RhoA^CKO^ mice were subjected to contextual fear conditioning 9 days after control- or CCI-injury. Freezing responses of mice were recorded before foot shock (Naive) and 24 hr after foot shock in the training chamber (Context A) or a novel chamber (Context B). All naive mice showed minimal freezing prior to training. After training, control RhoA^fl/fl^ mice exhibited more robust freezing behavior in Context A than B, whereas CCI-injured RhoA^fl/fl^ mice were unable to distinguish between the two contexts. In contrast, RhoA^CKO^ mice did not display any context discrimination deficit either before or after injury. N = 7–8 mice/genotype. Significance was determined by ANOVA followed by Tukey post-hoc analysis. Within a particular context (**A** or **B**), no significant difference was detected in freezing behavior between control and injured RhoA^fl/fl^ mice or control and injured RhoA^CKO^ mice. Figures show mean ± SEM, **p < 0.01, ***p < 0.001, ^§§§^p < 0.001.

To determine the effects of blocking neuronal RhoA signaling on recovery from TBI, 3–4 month old RhoA^fl/fl^ and RhoA^CKO^ mice were subjected to a controlled cortical impact (CCI) injury, a focal contusion model of TBI commonly used in rodents^30^. As expected, CCI delivered to the right parietal cortex caused cortical tissue loss from the ipsilateral cerebral hemisphere around the site of impact (Fig. 2A). The extent of cortical loss was determined by collecting mouse brains 14 days post-injury, sectioning and Nissl staining them, and then measuring cortical lesion size using ImageJ software^31^. We found that the CCI-injured RhoA^fl/fl^ and RhoA^CKO^ mice had similar contusion volumes (Fig. 2B), suggesting that the absence of neuronal RhoA failed to prevent neuronal cell death and degeneration at the site of injury.

We next examined whether neuronal RhoA deletion can mitigate TBI-induced motor dysfunction by subjecting RhoA^fl/fl^ and RhoA^CKO^ mice to CCI injury and then assessing their motor performance on an accelerating rotarod. The rotarod is a well-established, reliable test for measuring motor deficits and recovery in experimental rodent models of TBI^32, 33^. Mice were pre-trained on the rotarod and then tested for their latency to fall on days 0–5 post-injury. Both RhoA^fl/fl^ and RhoA^CKO^ mice displayed significant motor impairment on day 1 after injury (Fig. 2C), suggesting that neuronal deletion of *RhoA* does not alleviate the immediate effects of TBI on motor coordination. However, while the RhoA^fl/fl^ animals continued to display significant motor deficits on the rotarod for 3 days after TBI and only reached pre-TBI performance levels on day 4, the RhoA^CKO^ mice recovered their pre-TBI performance by day 2 (Fig. 2C). Thus, while ablating RhoA in neurons does not prevent TBI-induced cortical tissue loss, it does speed up recovery of motor function.

### TBI impairs contextual fear discrimination, which is restored by suppression of neuronal RhoA signaling

TBI often causes long-term cognitive impairments, including deficits in attention, problem solving, and spatial and working memory^32, 34^. TBI also affects fear memory in both humans and rodents, but the nature of these changes remains uncertain due to conflicting reports^35–40^. The ability of animals to form fear memories enables them to predict dangers in their environment, but can also result in anxiety disorders like post-traumatic stress disorder (PTSD) when dysregulated^41^. Both contextual fear memory and contextual fear discrimination (i.e. the ability to discriminate between a fearful and a non-fearful environment) depend on the hippocampus, which encodes contextual information, mediates memory formation, and is frequently damaged following TBI^41, 42^. To determine how TBI impacts contextual fear memory and context discrimination and whether blocking neuronal RhoA signaling reverses these effects, we performed control surgeries (anesthesia and incision) or CCI surgeries on adult RhoA^fl/fl^ and RhoA^CKO^ mice and then subjected them to contextual fear conditioning nine days later. Briefly, mice acclimatized to the conditioning chamber were assessed for baseline freezing behavior (Naive) and then exposed to two electric foot shocks (0.7 mA, 2-sec duration, 2-min interval) before returning to their home cage. To test contextual fear memory, mice were returned to the conditioning chamber (Context A) 24 hr after training and allowed to freely explore for 5 min while their freezing behavior was recorded. To test contextual fear discrimination, 2 hr later the mice were placed in a different chamber (Context B) for 5 min and their freezing behavior was recorded. Prior to fear conditioning, all mice exhibited minimal baseline freezing (Fig. 2D). However, 24 hr after fear conditioning, control RhoA^fl/fl^ and RhoA^CKO^ mice showed a clear discrimination between both contexts, i.e., they displayed robust freezing in Context A that was significantly greater than the freezing they exhibited in Context B (Fig. 2D). In contrast, CCI-injured RhoA^fl/fl^ mice froze an equivalent amount in both chambers (Fig. 2D), indicating a lack of contextual discrimination. Strikingly, CCI-injured RhoA^CKO^ mice exhibited significantly more freezing in Context A than Context B, similar to control RhoA^fl/fl^ and RhoA^CKO^ mice (Fig. 2D), suggesting that the contextual discrimination deficit caused by TBI is blocked by RhoA ablation. TBI did not appear to significantly alter the freezing behavior of mice in a particular context (e.g. the freezing behavior of control and CCI-injured RhoA^fl/fl^ mice in Context A was not significantly different), suggesting that contextual fear memory per se remained intact in these animals. Hence, TBI specifically disrupts contextual fear discrimination, impairing the ability of mice to distinguish between a fearful and a non-fearful environment, and RhoA deletion from neurons protects mice against this hippocampal-dependent fear memory deficit.

### Pharmacological inhibition of RhoA-ROCK signaling ameliorates motor and cognitive deficits after TBI

**Figure 3:**
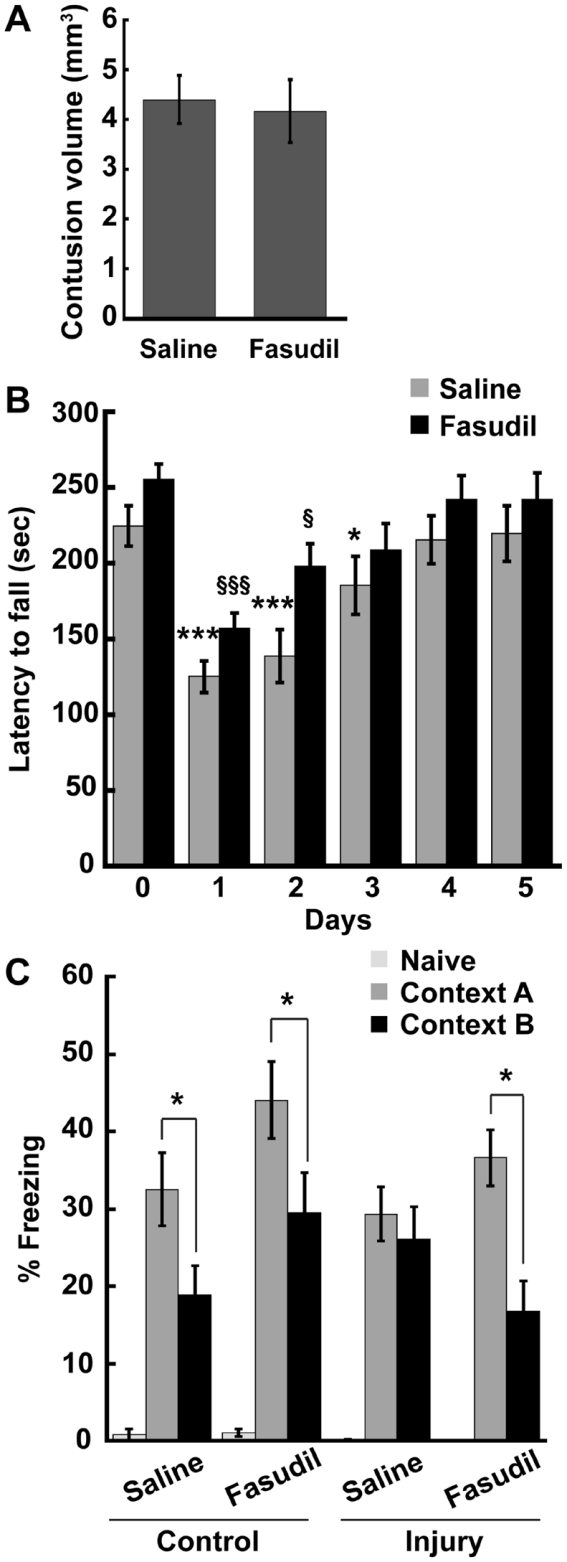
Fasudil treatment reverses motor and cognitive defects post-TBI. (**A**) Adult C57/Bl6 male mice were subjected to CCI-injury and then treated with saline or fasudil. Brains were collected 14 days post-injury and then sectioned, Nissl stained, and imaged. Contusion volumes were calculated using NIH ImageJ. Quantification of contusion volume revealed no difference between the two groups. Student’s t-test, N = 13–15 mice/group. (**B**) Pre-trained C57/Bl6 adult male mice were tested on an accelerating rotarod prior to CCI-injury (day 0) and for five consecutive days after injury and treatment with saline or fasudil. Fasudil-treated mice displayed a faster recovery of pre-TBI motor performance than saline-treated mice. Statistical significance was determined with two-way ANOVA with repeated measures, comparing pre-TBI performance to post-TBI performance for saline-treated mice (*) and fasudil-treated mice (§). N = 14 mice/group. (**C**) Control or CCI-injured C57/Bl6 mice were treated with saline or fasudil and then subjected to contextual fear conditioning 9 days after injury. Control saline- and fasudil-treated mice both froze more in Context A than Context B. In contrast, injured saline-treated mice were unable to discriminate between the two contexts, whereas fasudil treatment reversed this phenotype. N = 7–8 mice/group. Significance was determined by ANOVA followed by Tukey post-hoc analysis. Figures show mean ± SEM, *p < 0.05, ***p < 0.001, ^§^p < 0.05, ^§§§^p < 0.001.

Since genetic ablation of RhoA from postnatal forebrain neurons enhances recovery of motor and cognitive function after TBI, we next asked whether inhibiting RhoA signaling pharmacologically could achieve the same effect, as this type of strategy could lead to a potential treatment for TBI. To this end, we used fasudil since it successfully inhibits the key RhoA effector ROCK, and it has been proven safe for clinical use in Japan and China^19, 43–45^. Adult C57/Bl6 male mice were subjected to control or CCI injury and then 10 min post-injury, the mice were intraperitoneally administered vehicle (saline) or 10 mg/kg fasudil. These injections were continued once daily for the duration of the experiment. To determine whether pharmacological inhibition of RhoA-ROCK signaling affects the extent of focal tissue injury following TBI, we compared cortical contusion volumes between saline and fasudil treated mice 14 days post-injury. Consistent with our genetic data, we found that the lesion volumes were similar between saline and fasudil treated mice (Fig. 3A), suggesting that inhibiting RhoA-ROCK signaling does not enhance neuroprotection or repair at the site of injury.

Since RhoA^CKO^ mice displayed enhanced speed of recovery of motor function following TBI (Fig. 2C), we next examined whether ROCK inhibition affects motor performance post-injury. C57/Bl6 mice were trained on the accelerating rotarod on Day 0 and then subjected to a control or CCI surgery followed by treatment with saline or 10 mg/kg fasudil. Mice were tested on the rotarod for five successive days post-injury and their latency to fall was recorded. Consistent with our genetic analysis, mice performed poorly on the rotarod following TBI, and fasudil treatment sped recovery from this motor deficit (Fig. 3B). While saline-treated mice reached pre-TBI performance levels 4 days after TBI, fasudil-treated mice recovered motor function by 3 days post-injury. Thus, acute inhibition of RhoA-ROCK signaling attenuates the detrimental effects of TBI on motor coordination and balance.

To determine whether fasudil also enhances the recovery of cognitive function after TBI, we subjected control and CCI-injured C57/Bl6 mice treated with saline or fasudil to contextual fear conditioning beginning 9 days post-injury. As in Fig. 2D, we did not detect any significant change in the freezing behavior of either saline-treated or fasudil-treated mice in Context A as a result of TBI (Fig. 3C), and both saline- and fasudil-treated control mice were capable of discriminating between Context A and Context B (Fig. 3C). Importantly, we found that TBI induced a contextual fear discrimination defect in saline-treated mice, and this TBI-induced context discrimination deficit was reversed by fasudil treatment (Fig. 3C). These findings confirm that inhibiting the RhoA-ROCK pathway prevents hippocampal-dependent contextual fear discrimination impairments following brain injury.

### RhoA-ROCK inhibition blocks TBI-induced dendritic spine remodeling

**Figure 4:**
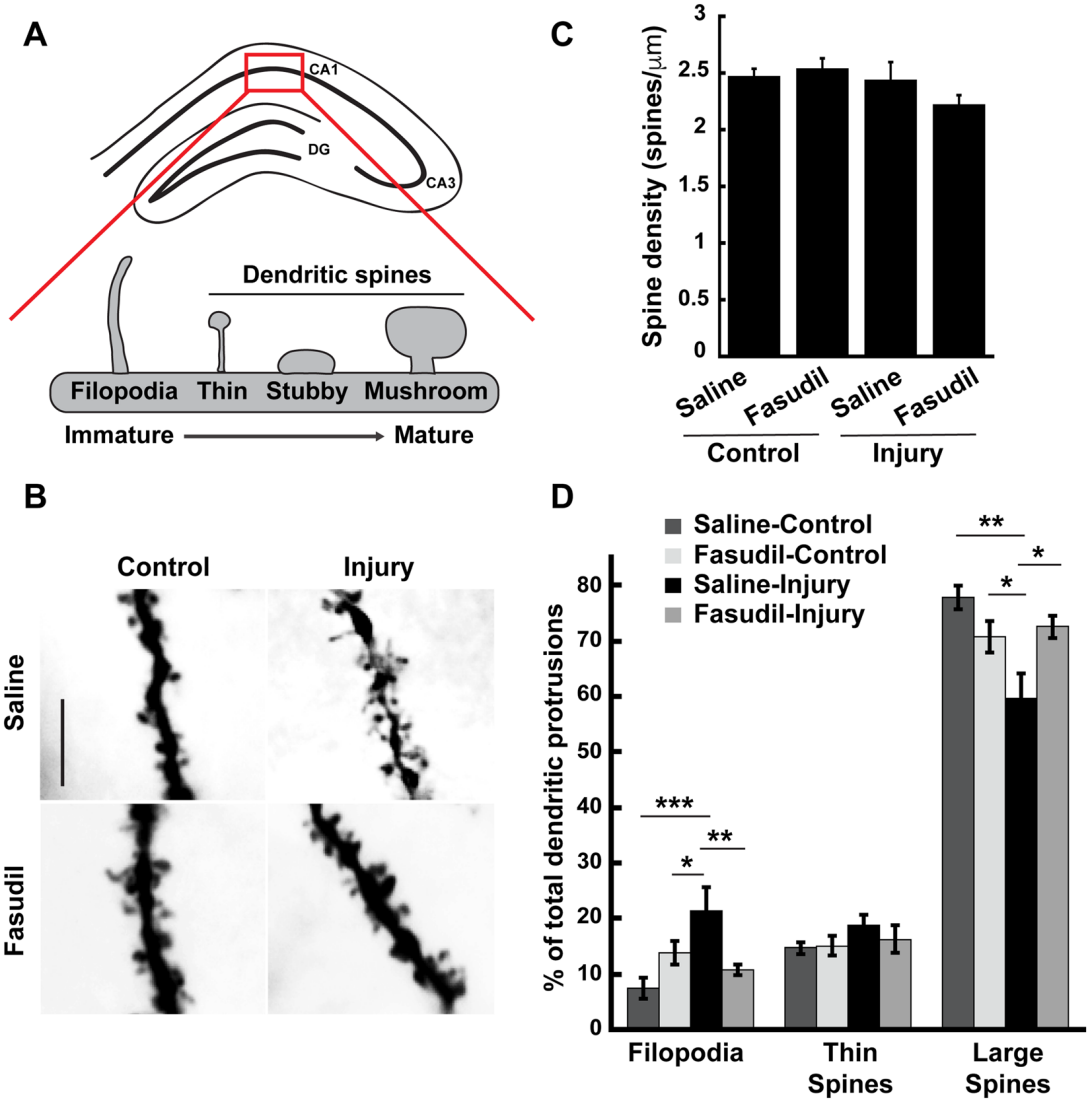
Blocking RhoA-ROCK signaling with fasudil prevents TBI-evoked abnormal dendritic spine remodeling. (**A**) Dendrites for spine analysis were selected from Golgi-stained pyramidal neurons from area CA1 of the hippocampus (marked by the red box) ipsilateral to the injury. Dendritic protrusions vary in shape and size from long, thin, immature filopodia, which may or may not be associated with a synapse, to large, mature mushroom-shaped spines, which are typically associated with strong synapses. (**B**) Brains from control or CCI-injured mice treated with saline or fasudil were collected 3 days after surgeries and processed for Golgi staining. High magnification images of CA1 hippocampal pyramidal neurons were used to quantify the density of different dendritic protrusions. Scale bar = 5 μm. (**C**) Quantification of the total spine density of dendritic protrusions. No significant differences were observed between the different groups. N = 7–8 mice/group. (**D**) CCI-injury induced an increase in the percentage of immature filopodia and a decrease in the percentage of large (stubby and mushroom) spines on neurons from saline-treated mice. Fasudil treatment blocked these CCI-elicited structural modifications. N = 7–8 mice per group. Figures show mean ± SEM, ANOVA followed by Tukey post hoc analysis, *p < 0.05, **p < 0.01, ***p < 0.001.

How might inhibiting RhoA-ROCK signaling enhance recovery after TBI? Since ablating RhoA specifically in postnatal forebrain neurons (i.e. in RhoA^CKO^ mice) is sufficient to expedite restoration of motor and cognitive function, RhoA signaling in neurons is likely to be at least partially responsible for the TBI-induced deficits. Neuronal RhoA-ROCK signaling could cause functional impairments by promoting the retraction and/or loss of dendritic spines^46–49^. Spines are small, actin-rich protrusions on dendrites that serve as the primary sites for excitatory synaptic input in the brain^50–52^. Spines can be classified into different morphological categories; filopodia (spine precursors, long and thin with no spine head), thin spines (long thin neck and small bulbous head), stubby spines (no neck), and mushroom spines (short thin neck and large bulbous head) (Fig. 4A). Spine morphology is highly correlated with spine stability and synaptic strength, with mushroom spines being the most stable and containing the largest, strongest synapses^53^. Spines rapidly remodel in response to neural activity, which is critical for neural circuit development, synaptic plasticity, and learning and memory^54^. In contrast, abnormal spine morphogenesis, commonly associated with brain disorders and injury, is thought to impair information processing and memory storage^16, 55–57^. To determine whether inhibiting RhoA-ROCK signaling prevents TBI-induced spine remodeling, we treated control and CCI-injured C57/Bl6 mice with saline or fasudil for 3 days, and then mouse brains were collected and subjected to Golgi staining to visualize neuron morphology. Golgi-stained dendrites on CA1 hippocampal pyramidal neurons were imaged and analyzed for spine density and morphology (Fig. 4B). While we detected no significant decrease in total spine density on CA1 pyramidal neurons following TBI (Fig. 4C), we did observe a substantial reduction in the density of large spines (mushroom-shaped and stubby spines) and a corresponding increase in filopodia density in saline-treated injured mice as compared to saline- or fasudil-treated control mice (Fig. 4B,D). These results indicate that TBI induces spine remodeling on CA1 pyramidal neurons that involves the retraction and/or loss of large, mature spines and the formation of new, immature filopodia. Since large spines typically house bigger, stronger synapses, while filopodia often lack associated synapses, TBI-induced spine remodeling likely causes a loss of established synaptic connections and the formation of new abnormal connections that could underlie the functional deficits (e.g. contextual discrimination memory defect) observed after TBI. Importantly, fasudil treatment of CCI-injured mice restored the density of large spines and filopodia to control levels (Fig. 4B,D), suggesting that RhoA-ROCK inhibition prevents TBI-induced pathological spine remodeling.

## Discussion

In this study, we showed that TBI causes motor and cognitive impairments in mice that are alleviated by genetically or pharmacologically blocking RhoA-ROCK signaling. Specifically, we found that mice subjected to TBI perform poorly on an accelerating rotarod, displaying transient deficits in motor coordination and balance. Motor function is typically mediated by a complex system of neural networks involving the primary and secondary motor cortices, with inputs from the cerebellum, brainstem, and subcortical nuclei^33^. TBI-induced deficits in motor function and sensorimotor integration are thought to arise from disruptions in these complex neural pathways^33^. Notably, inhibition of RhoA-ROCK signaling accelerates restoration of normal motor function, perhaps by protecting or enhancing repair of these motor-associated brain circuits. Likewise, we found that TBI disrupts contextual fear discrimination in mice, leading to an overgeneralization of fear reminiscent of PTSD-like symptoms, frequently found in individuals with TBI^58^. The underlying cause of this contextual discrimination impairment may be a defect in pattern separation, a hippocampal-dependent process that helps to distinguish between two similar experiences and ensures that memories are stored independently from one another^59–63^. Importantly, we found that blocking RhoA-ROCK signaling abolishes the contextual fear discrimination impairment we detected in TBI-injured mice. Overall, irrespective of how we inhibited RhoA-ROCK signaling (genetically or pharmacologically), we obtained similar results. The minor differences in functional recovery we observed between our genetic and pharmacological experiments could be explained by differences in the genetic backgrounds of the mice (mixed vs. C57/Bl6) and/or the cell type in which RhoA-ROCK signaling was suppressed (neurons vs. all cell types). Regardless, both studies clearly demonstrate that inhibiting RhoA-ROCK signaling enhances functional recovery from TBI.

In an attempt to identify how blocking RhoA-ROCK signaling promotes functional recovery after TBI, we found that while it fails to impede cortical tissue loss, it prevents TBI-induced dendritic spine remodeling and mature spine loss on hippocampal pyramidal neurons. A similar effect may also occur on neurons from motor-associated brain regions. Since spines are the main sites of excitatory synaptic transmission in the brain and spine structure is strongly correlated with synaptic function^9^, it is likely that TBI-induced spine remodeling contributes to impaired information processing in the brain, and blocking this remodeling attenuates cognitive/motor functional deficits. Spines are highly enriched in filamentous actin (F-actin), and their capacity to change shape relies on the rapid rearrangement of the actin cytoskeleton within spines. Rho-family GTPases orchestrate the actin cytoskeletal dynamics that drive the formation and remodeling of spines^64^. While Rac1 and Cdc42 generally promote spine formation and growth, the activation of RhoA results in spine retraction and loss^9^. The serine/threonine kinase ROCK mediates the effects of RhoA through the phosphorylation and regulation of multiple downstream targets, including myosin light chain (MLC), which promotes actomyosin-based contractility, and LIM-kinase (LIMK), which phosphorylates and inactivates the actin-depolymerizing factor cofilin^65^. Since treatment of mice with the ROCK inhibitor fasudil blocks the TBI-elicited increase in immature filopodia and loss of mature spines on hippocampal pyramidal neurons, it is likely that one or more of these RhoA-ROCK signaling pathways is responsible for TBI-induced spine remodeling.

Consistent with our fasudil results, a recent report by Bye *et al*. showed that continuous infusion of the ROCK inhibitor Y27632 into the lateral ventricle of mice improved motor function after TBI (i.e. fewer foot faults on a horizontal ladder), but did not promote tissue sparing^66^. The authors did not investigate whether Y27632 infusion also blocks TBI-induced cognitive deficits or spine remodeling. Nevertheless, both studies support the hypothesis that pharmacologically targeting RhoA-ROCK signaling enhances recovery from TBI. Since neither drug treatment was exclusive for neurons, it is possible that inhibiting RhoA-ROCK signaling in non-neuronal cells contributed to the enhanced recovery response to TBI. Indeed, blocking RhoA-ROCK signaling in glia is known to attenuate their responses to injury^67–73^. However, the overall similarity between our results with fasudil-treated mice and RhoA^CKO^ mice, in which RhoA was genetically ablated specifically from forebrain neurons, argues that RhoA-ROCK signaling in neurons is an important target for promoting functional recovery from TBI. Moreover, Bye *et al*. found that Y27632 treatment had no effect on TBI-induced microglial accumulation or astrocytic gliosis 7 or 35 days post-injury^66^. Nonetheless, given the important roles RhoA-ROCK signaling plays in glia, the effects of RhoA-ROCK inhibition on non-neuronal cell types including astrocytes and microglia following TBI require further investigation.

Collectively, our findings suggest that TBI elicits pathological spine remodeling that likely contributes to behavioral deficits due to loss and/or alteration of synaptic connections. Moreover, inhibiting RhoA-ROCK signaling enhances functional recovery, at least in part, by protecting against this detrimental effect. Thus, targeting RhoA-ROCK signaling is an effective therapeutic strategy for treating TBI-induced deficits. As fasudil has been used safely in humans in Japan and China to treat cerebral vasospasms and ischemic stroke^19, 44^, our results suggest that it might be an invaluable drug for the treatment of TBI, particularly if used in combination therapy with other neuroprotective and/or anti-inflammatory drugs.

## Experimental Procedures

### Animals

All mice were housed with free access to food and water ad libitum and maintained in a 12/12 hr light/dark cycle. RhoA^fl/fl^ mice, generated as described^23^ were crossed with RhoA^fl/+^; *CamKIIα-Cre* (RhoA het) mice to obtain RhoA^fl/fl^; *CamKIIα-Cre* (RhoA^CKO^) as well as RhoA het and RhoA^fl/fl^ (control) littermates for experiments. RhoA^fl/fl^ mice were maintained on a 129SvEv background, while *CamKIIα-Cre* mice were maintained on a C57/Bl6 background. Genotyping was performed by polymerase chain reaction (PCR). All animal experiments were carried out in strict accordance with the recommendations provided by the Guide for the Care and Use of Laboratory Animals of the National Institutes of Health and were approved by the Baylor College of Medicine Institutional Animal Care and Use Committee. All efforts were made to minimize animal suffering.

### Controlled Cortical Impact

Adult (3–4 month old) male C57BL/6, RhoA^fl/fl^ (control), and RhoA^CKO^ mice of similar weight were subjected to a moderate controlled cortical impact (CCI) or control injury as previously described, with some modifications^16, 74^. Briefly, mice were anesthetized under 4% Isoflurane and placed in a stereotaxic apparatus (Kopf Instruments, Tujunga, CA) with a continuous supply of 2% Isoflurane through a nosepiece. Using sterile procedures, an incision was made along the midline of the scalp, followed by a unilateral 2 mm craniotomy between the Bregma and Lambda. Without disturbing the dura underneath, the bone flap was carefully removed to expose the cortical surface. Mice were then subjected to CCI using an impactor device (Leica Biosystems) with a 3.0 m/sec piston velocity and a 2.0 mm deformation impact (injured mice). To avoid craniotomy-induced brain damage, control mice were subjected only to skin incisions that were sutured back under anesthesia^75–78^. After surgeries, mice were returned to their home cage and monitored for recovery. Mice were subcutaneously injected twice daily for 3 days with 1 mg/kg Buprenorphine as an analgesic. Fasudil-treated C57BL/6 mice were intraperitoneally injected with 10 mg/kg fasudil hydrochloride [5-(1,4-Diazepane-1-sulfonyl) isoquinoline, (Tocris Bioscience, USA)] for the indicated number of days, while saline-treated C57BL/6 mice were injected with saline for the equivalent time. The first dose was administered 10 min after monitoring the mice for postoperative recovery.

### Rotarod

To examine baseline motor behavior, naive RhoA^fl/fl^ (control) and RhoA^CKO^ mice (11 adult male mice/genotype) were subjected to an accelerating rotarod test on two consecutive days with four trials/day. Mice rested at least 30 min between trials. The rotation speed of the rotarod increased from 4 to 40 rpm during the test. The duration of time the mice stayed on the rotarod (latency to fall) was recorded in seconds and all 8 trials were analyzed. To study the effect of TBI on motor performance, adult male mice were separated into two groups per experiment (8–14 mice/group); CCI-injured RhoA^fl/fl^ and RhoA^CKO^ mice (for genetic study), or CCI-injured saline-treated and fasudil-treated (for fasudil study). The experimenter was blinded to the genotype and treatment. Mice in each group were pre-trained on the rotarod prior to surgery (Day 0). A baseline value of latency to fall was determined for each mouse, and trained mice performing worse than 100 s on day 0 were excluded from the study. Following training, mice were subjected to CCI surgery and then further assessed for motor performance on days 1, 2, 3, 4 and 5 post-surgery. On each assessment day, mice were given two trials for a maximum of 5 min each and their latency to fall was recorded. To compare differences in the performance over time of control and experimental mouse groups, the data were analyzed using two-way ANOVA with repeated measures. For each individual mouse group, we also used one-way repeated measures ANOVA to identify time points where mice performed significantly worse on the rotarod than on day 0 (prior to injury).

### Context Discrimination Test

To examine baseline cognitive function, RhoA^fl/fl^ and RhoA^CKO^ adult male mice were subjected to a fear-conditioning paradigm. After 2 min of exploration in the training chamber, mice received 2 pairings of a 30 s tone (2800 Hz, 85 dB, spaced by 1 min) and a co-terminating electrical shock (0.7 mA, 2 s). To test contextual fear memory, 24 hr later, animals were returned to the conditioning chamber for 5 min and their freezing percentages were calculated. Data were compared using ANOVA followed by Tukey post hoc analysis. To study the effect of TBI on fear memory and context discrimination, control and CCI-injured mice (RhoA^fl/fl^, RhoA^CKO^, saline-treated, or fasudil-treated) received fear-conditioning training on day 9 after surgery. For conditioning, mice were allowed 2 min of free exploration in the training chamber (Context A) before receiving 2 pairings of a 30 s tone and a co-terminating electrical shock. 24 hr later, animals were returned to the conditioning chamber (Context A) and allowed to explore freely with no shock or tone for 5 min while their freezing responses were recorded. To test contextual discrimination, 2 hr later, mice were placed in a different chamber (Context B) for 5 min and again their freezing responses were recorded. In all cases, the experimenter was blinded to the genotype or treatment groups. The whole procedure was videotaped and freezing percentages were calculated using FreezeFrame software (Coulborn Instruments, MA). 7–8 adult mice were used per group. Data from the contextual fear conditioning experiments were compared using ANOVA followed by Tukey post hoc analysis to compare % freezing in Context A versus Context B across the different groups, and to identify differences in freezing behavior between the different mouse groups in one particular context (Context A or B).

### Nissl Staining and Contusion Volume Determination

Mice were anesthetized with isoflurane and transcardially perfused with Phosphate Buffered Saline (PBS) followed by 4% Paraformaldehyde (PFA). Brains were collected, sectioned, and stained with a 0.1% cresyl violet solution. The sections were then mounted in Cytoseal 60 (Richard Allan Scientific, MI). For contusion volume determination, brains were collected at 14 days post-injury and 30 µm coronal sections were mounted on slides. At 0.27 mm intervals, four sections were obtained, stained with cresyl violet and imaged. Lesions from the injured cortex were traced and the area was quantified using ImageJ. From these data, corresponding lesion volumes were calculated and were expressed in mm^3^. Data was collected from 13–15 mice/group and were compared using Student’s t-test.

### Golgi Staining and Spine Quantification

The FD Rapid Golgi Stain kit (FD NeuroTechnologies) was used to perform Golgi staining. Briefly, freshly dissected brains were quickly rinsed in distilled water, transferred to impregnation solution (equal volumes of Solutions A and B), and stored in the dark at room temperature for around 2 weeks. The brains were then immersed into solution C and kept in the dark for 72 hrs at 4 °C. 50 µm brain sections were collected from the injury site between Bregma and Lambda. Following Golgi staining, we collected images using 40X magnification (Zeiss upright microscope) from two sections that were −2.0 and −2.1 mm from Bregma, with the experimenter blinded to the sample conditions. To analyze spine density and morphology, 2–3 apical dendrites/section were selected from hippocampal pyramidal neurons in the CA1 region ipsilateral to the injury site, with a total of 5–6 dendrites/mouse imaged. Data were collected from 7–8 mice per experimental group. Spine density was calculated as spines/µm dendrite length. To assess the TBI-induced changes in spine morphology, spines on selected dendrite segments were classified in a blinded manner as mushroom, stubby, thin or filopodia. The following criteria were used: mushroom spine - large bulbous head with a short neck; thin spine - small head and slender long neck; stubby spine - head with no neck; and filopodia - long protrusions with no head. Mushroom and stubby spines were combined into one category called ‘large spines’ to avoid overestimating stubby spines and underestimating mushroom spines since short-necked, mushroom-shaped spines frequently appear stubby using traditional light microscopy due to its lower optical resolution^79^. Spine densities and percentages of spines in each category were compared using ANOVA followed by Tukey post hoc analysis.

### Protein Lysates and Western Blot Analysis

Hippocampal and cortical tissue from RhoA^fl/fl^ and RhoA^CKO^ mice were homogenized in RIPA lysis buffer (10 mM Tris-Cl pH 8.0, 1 mM EDTA, 0.5 mM EDTA, 1% TritonX-100, 0.1% sodium deoxycholate, 0.1% SDS, 140 mM NaCl) using a Dounce homogenizer. Protein amounts were estimated using the Bicinchoninic acid assay (BCA assay, Thermo Fisher Scientific, Waltham, MA) according to the manufacturer’s protocol. Protein lysates were separated using sodium dodecyl sulfate (SDS) – polyacrylamide gel electrophoresis and transferred onto PVDF membranes. The membranes were incubated in blocking buffer [5% skimmed milk powder in 50 mM Tris, pH 8.0, 150 mM NaCl, 0.05% Tween (TBST)] followed by an overnight incubation with primary antibody at 4 °C. Rabbit polyclonal primary antibodies anti-RhoA, (Cell Signaling Technology, Beverly, MA) and anti-GAPDH (Santacruz Biotechnologies Inc, Dallas, TX) and anti-rabbit goat polyclonal secondary antibodies labeled with horseradish peroxidase (Cell signaling Technology, Beverly, MA) were used in this study. The secondary antibody binding was detected by enhanced chemiluminescence (ECL, Thermo Fisher Scientific, Waltham, MA) using Image quant LAS400 (GE Healthcare Lifesciences, PA).

### Statistical Analysis

All statistical parameters were calculated using KaleidaGraph software (Synergy Software, Reading, PA). The collected data were expressed as mean ± standard error (SEM) and differences were analyzed using Student’s t-test or ANOVA followed by Tukey post hoc analysis unless otherwise mentioned. *P* < 0.001, 0.01, or 0.05 was considered significant.

## Electronic supplementary material

S1
