# Supplementary material for: RhoA-ROCK Inhibition Reverses Synaptic Remodeling and Motor and Cognitive Deficits Caused by Traumatic Brain Injury
Source: Sci Rep. 2017 Sep 6;7:10689. doi: 10.1038/s41598-017-11113-3 (PMC5587534; doi:10.1038/s41598-017-11113-3)
Supplement: Supplementary file 1 — S1 [file 41598_2017_11113_MOESM1_ESM.docx]

**RhoA-ROCK Inhibition Reverses Synaptic Remodeling and Motor and Cognitive Deficits Caused by Traumatic Brain Injury**

Shalaka Mulherkar^1#^, Karen Firozi^1#^, Wei Huang^1^*, Mohammad Danish Uddin^1^, Raymond J. Grill^3^**, Mauro Costa-Mattioli^1,2^, Claudia Robertson^4^, Kimberley F. Tolias^1,5,§^

^1^Department of Neuroscience, Baylor College of Medicine, Houston, TX 77030

^2^Memory and Brain Research Center, Baylor College of Medicine, Houston, TX 77030

^3^Department of Integrative Biology and Pharmacology, University of Texas Medical School at Houston, TX 77030

^4^Department of Neurosurgery, Baylor College of Medicine, Houston, TX 77030

^5^Verna and Marrs McLean Department of Biochemistry and Molecular Biology, Baylor College of Medicine, Houston, TX 77030.

*Present address: The Solomon Snyder Department of Neuroscience, Johns Hopkins University School of Medicine, 733 N. Broadway, Baltimore, Maryland 21205, USA

**Present address: Department of Neurobiology and Anatomical Sciences, University of Mississippi Medical Center, Jackson, MS, 39216

^§^Corresponding author: Kimberley F. Tolias, Ph.D, Baylor College of Medicine, One Baylor Plaza, M.S. BCM 295, Houston, TX 77030, tolias@bcm.edu

^#^These authors contributed equally to the study.

**
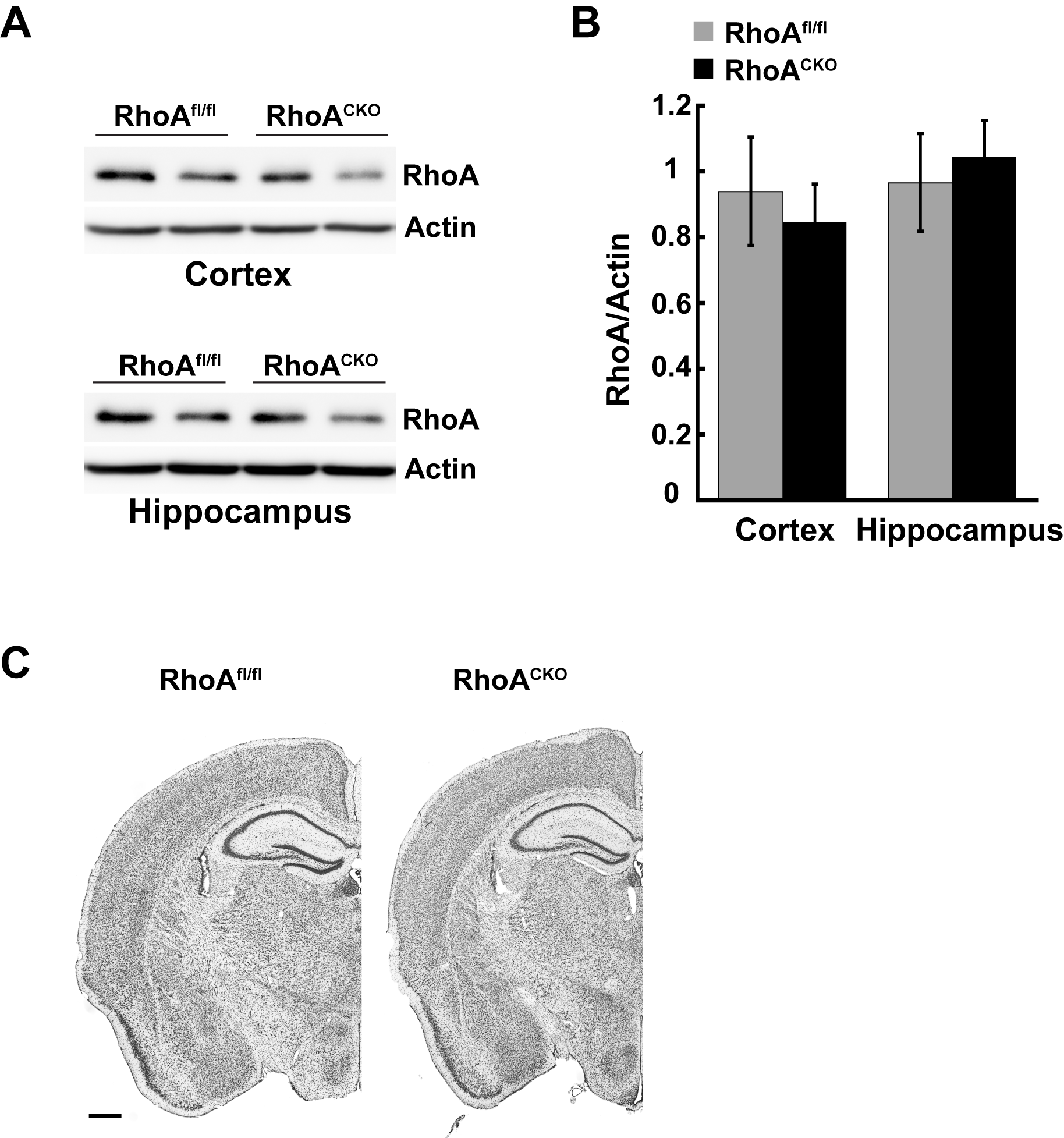
**

**Figure S1.** (A) Cortical and hippocampal lysates from 1-month old RhoA^fl/fl^ and RhoA^CKO^ mice were immunoblotted for RhoA or actin (loading control). Representative blots show no changes in RhoA levels in RhoA^fl/fl^ and RhoA^CKO^ mice at this age. (B) Quantification of RhoA protein levels in control and RhoA^CKO^ mice. Protein bands were quantified using NIH Image J software and normalized using actin as control. (N=3 mice/genotype, Student’s t-test). (C) Representative Nissl stained coronal brain sections from adult RhoA^fl/fl^ and RhoA^CKO^ mice reveal normal brain structures in RhoA^CKO^ mice (N=3). Scale bar= 500 μm.
